# Selective vulnerability of Rich Club brain regions is an organizational principle of structural connectivity loss in Huntington’s disease

**DOI:** 10.1093/brain/awv259

**Published:** 2015-09-18

**Authors:** Peter McColgan, Kiran K. Seunarine, Adeel Razi, James H. Cole, Sarah Gregory, Alexandra Durr, Raymund A. C. Roos, Julie C. Stout, Bernhard Landwehrmeyer, Rachael I. Scahill, Chris A. Clark, Geraint Rees, Sarah J. Tabrizi

**Affiliations:** 1 Department of Neurodegenerative Disease, UCL Institute of Neurology, London, WC1N 3BG, UK; 2 Developmental Imaging and Biophysics Section, UCL Institute of Child Health, London, WC1N 1EH, UK; 3 Wellcome Trust Centre for Neuroimaging, UCL Institute of Neurology, London, WC1N 3BG, UK; 4 Department of Electronic Engineering, NED University of Engineering and Technology, Karachi, Pakistan; 5 Computational, Cognitive and Clinical Neuroimaging Laboratory, Department of Medicine, Imperial College London, W12 0HS, UK; 6 APHP Department of Genetics, Groupe Hospitalier Pitié-Salpêtrière, and Institut du Cerveau et de la Moelle, INSERM U1127, CNRS UMR7225, Sorbonne Universités – UPMC Université Paris VI UMR_S1127, Paris, France; 7 Department of Neurology, Leiden University Medical Centre, 2300RC Leiden, The Netherlands; 8 School of Psychological Sciences, Monash University, VIC, Australia; 9 Department of Neurology, University of Ulm, Oberer Eselsberg 45-1, D-89081, Ulm, Germany; 10 National Hospital for Neurology and Neurosurgery, Queen Square, London, WC1N 3BG, UK

**Keywords:** tractography, rich club, Huntington’s disease

## Abstract

Huntington’s disease can be predicted many years before symptom onset, and thus makes an ideal model for studying the earliest mechanisms of neurodegeneration. Diffuse patterns of structural connectivity loss occur in the basal ganglia and cortex early in the disease. However, the organizational principles that underlie these changes are unclear. By understanding such principles we can gain insight into the link between the cellular pathology caused by mutant huntingtin and its downstream effect at the macroscopic level. The ‘rich club’ is a pattern of organization established in healthy human brains, where specific hub ‘rich club’ brain regions are more highly connected to each other than other brain regions. We hypothesized that selective loss of rich club connectivity might represent an organizing principle underlying the distributed pattern of structural connectivity loss seen in Huntington’s disease. To test this hypothesis we performed diffusion tractography and graph theoretical analysis in a pseudo-longitudinal study of 50 premanifest and 38 manifest Huntington’s disease participants compared with 47 healthy controls. Consistent with our hypothesis we found that structural connectivity loss selectively affected rich club brain regions in premanifest and manifest Huntington’s disease participants compared with controls. We found progressive network changes across controls, premanifest Huntington’s disease and manifest Huntington’s disease characterized by increased network segregation in the premanifest stage and loss of network integration in manifest disease. These regional and whole brain network differences were highly correlated with cognitive and motor deficits suggesting they have pathophysiological relevance. We also observed greater reductions in the connectivity of brain regions that have higher network traffic and lower clustering of neighbouring regions. This provides a potential mechanism that results in a characteristic pattern of structural connectivity loss targeting highly connected brain regions with high network traffic and low clustering of neighbouring regions. Our findings highlight the role of the rich club as a substrate for the structural connectivity loss seen in Huntington’s disease and have broader implications for understanding the connection between molecular and systems level pathology in neurodegenerative disease.

## Abstract (precis)

Diffuse structural connectivity loss occurs early in Huntington’s disease. However, the organizational principles underlying these changes are unclear. Using whole brain diffusion tractography and graph theoretical analysis, McColgan, Seunarine *et al.* identify a specific role for highly connected rich club regions as a substrate for structural connectivity loss in Huntington’s disease.

## Graphical abstract

**Figure:**
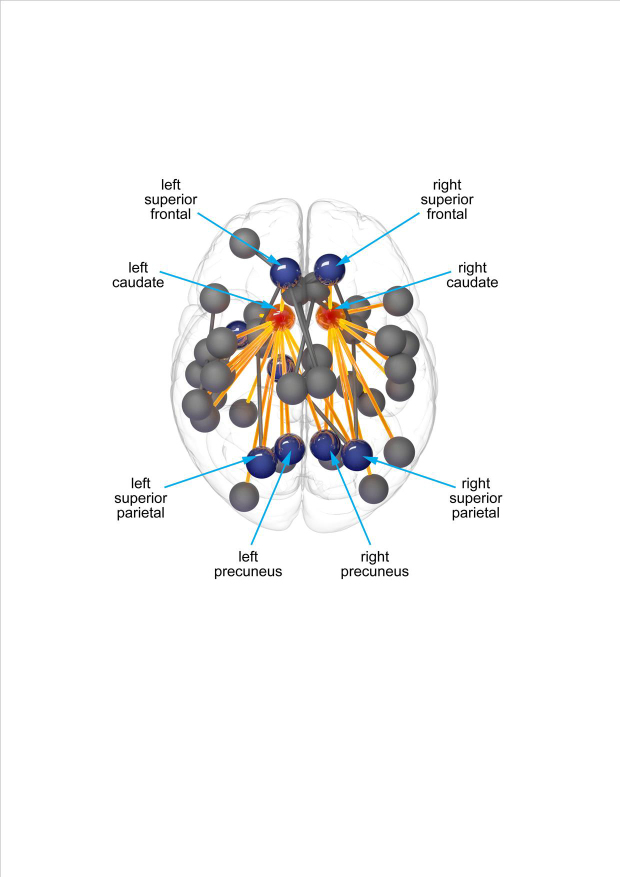
Diffuse structural connectivity loss occurs early in Huntington’s disease. However, the organizational principles underlying these changes are unclear. Using whole brain diffusion tractography and graph theoretical analysis, McColgan, Seunarine *et al.* identify a specific role for highly connected rich club regions as a substrate for structural connectivity loss in Huntington’s disease.

## Introduction

Huntington’s disease is an autosomal dominant neurodegenerative disorder caused by a CAG repeat expansion in the *HTT* gene on chromosome 4. The full penetrance of Huntington’s disease in mutation carriers with >39 CAG repeats makes it a model for studying the preclinical phase of neurodegeneration, as it is possible to predict who will develop the disease many years before symptom onset. Loss of caudate volume and surrounding white matter occur early in the premanifest stage, while more extensive grey and white matter loss, extending to cortical regions is seen in manifest disease (Tabrizi *et al.*, 2011). Consistent with this grey and white matter loss, recent investigations using diffusion tensor imaging tractography reveal loss of structural connectivity between the basal ganglia and cortex (Kloppel *et al.*, 2008; Bohanna *et al.*, 2011; Marrakchi-Kacem *et al.*, 2013; Novak *et al.*, 2015) and across a diffuse cortical network (Poudel *et al.*, 2014). However, such observational studies have not yet revealed whether there are any organizational principles that underlie this distributed pattern of structural connectivity loss. Understanding whether such principles exist will provide insight into the link between the cellular pathology of Huntington’s disease and its effect at the level of cortical organization affecting particular structures and networks.

The ‘rich club’ is one such pattern of organization established in healthy human brains. Structural connections are not uniform but are organized across the brain in a non-homogenous fashion. Specific hub (‘rich club’) brain regions are more highly connected to each other, forming a selective network with higher connectivity than other brain regions (van den Heuvel and Sporns, 2011). Cortical rich club regions include the superior frontal, superior parietal, precuneus and insula and are reproducible across studies (van den Heuvel and Sporns, 2011; van den Heuvel *et al.*, 2013). Such topological centrality of the rich club network supports integrative processing and adaptive behaviours (Senden *et al.*, 2014). Consistent with this, the degree of structural rich club connectivity predicts general cognitive performance in healthy older adults (Baggio *et al.*, 2015).

We hypothesized that selective loss of rich club connectivity might represent an organizing principle underlying the distributed pattern of structural connectivity loss seen in Huntington’s disease. Such a hypothesis, if correct, would be consistent with the trans-neuronal spread of pathogenic misfolded proteins that are a feature of Huntington’s disease (Pecho-Vrieseling *et al.*, 2014) and other neurodegenerative diseases, such as Alzheimer’s (Braak and Braak, 1998; Braak and Del Tredici, 2011; Yin *et al.*, 2014) and Parkinson’s disease (Braak *et al.*, 2003; Olanow and Brundin, 2013). This trans-neuronal spread would be expected to selectively target highly connected hub or rich club regions thus linking a general cellular pathology to a selective topological targeting of specific brain areas.

Using diffusion tractography and graph theoretical analysis in premanifest and manifest Huntington’s disease and healthy individuals we set out to test the following hypotheses: (i) Huntington’s disease leads to selective structural connectivity loss of rich club regions causing breakdown of the whole brain network; (ii) such regional rich club and whole brain network changes are associated with cognitive and motor deficits seen in Huntington’s disease; and (iii) highly connected brain regions with high network traffic and low clustering of neighbouring regions are more susceptible to structural connectivity loss.

## Materials and methods

### Cohort

We studied a cohort including Huntington’s disease (*n = *38), premanifest Huntington’s disease (*n = *50) and control participants (*n = *47) from the London, Paris and Leiden sites of the TRACK-Huntington’s disease study (Supplementary Table 1). Premanifest gene carriers required a disease burden score > 250, based on their medical records at the time of assessment, and a UHDRS total motor score ≤ 5, indicating no substantial motor signs. Controls were selected from the spouses or partners of individuals with premanifest or early Huntington’s disease or were gene-negative siblings, to ensure consistency of environments. Additional inclusion and exclusion criteria are detailed elsewhere (Tabrizi *et al.*, 2009). Statistical analysis was carried out using SAS version 9.4. The study was approved by the local ethics committees, and written informed consent was obtained from each participant. Left-handed participants were excluded from the analyses to avoid confounding effects caused by differences in structural connectivity in those who are right hemisphere dominant.

### MRI acquisition

T_1_- and diffusion-weighted images were acquired on Siemens (London and Paris) and Philips (Leiden) 3 T MRI scanners. Scanning time was ∼ 10 min for T_1_-weighted and 9 min for diffusion-weighted acquisitions. Detailed information on acquisition and head coil parameters are provided in the Supplementary material.

### Preprocessing

Cortical and subcortical regions of interest were generated by segmenting a T_1_-weighted image using Freesurfer (Desikan *et al.*, 2006). These included 70 cortical regions and six subcortical regions (caudate, putamen and thalamus bilaterally). Cortical and subcortical parcellations were visually reviewed for each subject for quality control. The globus pallidus and nucleus accumbens were excluded as regions of interest due to poor segmentations. To ensure the exclusion of these regions did not affect our results an 80-region graph theory analysis including these regions is provided in the Supplementary material. These targets were then warped into diffusion space by finding the mapping between the T_1_-weighted image and fractional anisotropy map using the NiftyReg toolkit (Modat *et al.*, 2010) and applying the resulting warp to each of the regions of interest.

The Freesurfer segmentation was also used to generate foreground masks for tractography. The graph analysis uses a foreground mask generated by combining the cortical/subcortical grey matter masks with the white matter mask. For the corticobasal ganglia connectivity analysis [including voxel connectivity profiles (VCPs)], two foreground masks were generated, one for the left hemisphere and the other for the right hemisphere, allowing investigation of intrahemispheric connectivity for each basal ganglia region.

**Figure 1:**
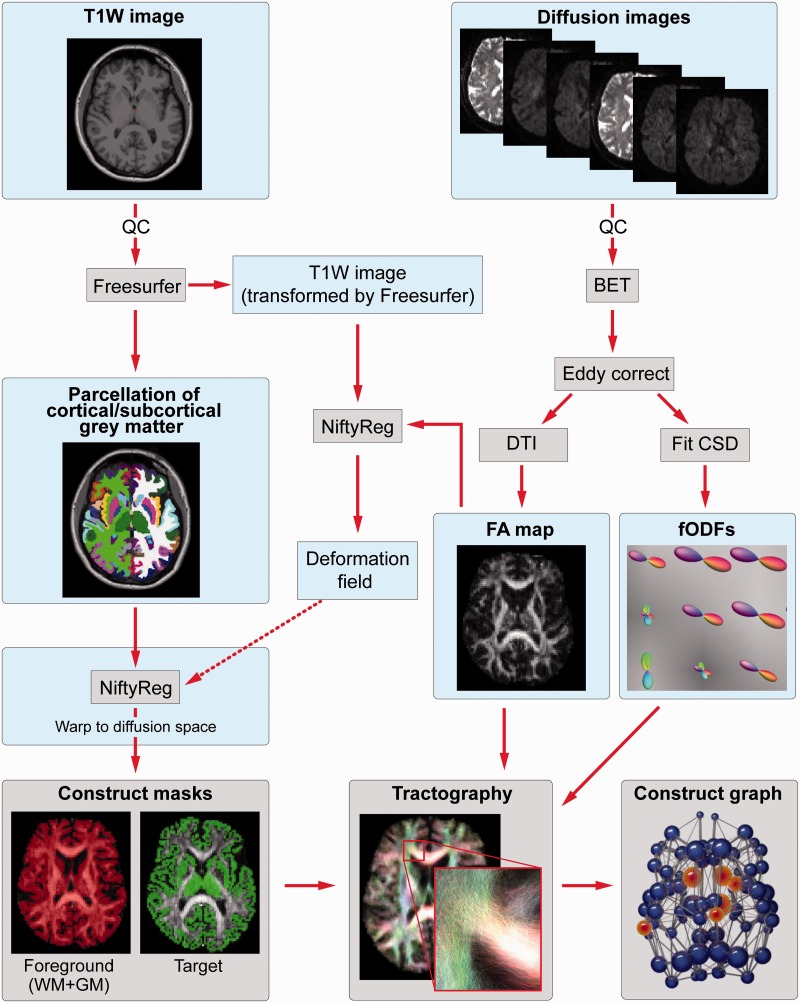
**Summary of processing pipeline**. BET = Brain Extraction Tool; CSD = constrained spherical deconvolution; DTI = diffusion tensor imaging; FA = fractional anisotropy; fODF = fibre orientation distribution function; GM = grey matter; QC = quality control; WM = white matter.

Diffusion data were preprocessed as follows: first the b = 0 image was used to generate a brain mask using FSL’s brain extraction tool (Smith, 2002). This mask was then eroded by one voxel to provide a more stringent mask. Next, eddy correct was used to align the diffusion-weighted volumes to the first b = 0 image and the gradient directions updated to reflect the changes to the image orientations. Finally, data were reconstructed using diffusion tensor imaging and constrained spherical deconvolution (CSD), as implemented in MRtrix (Tournier *et al.*, 2012). CSD was used as it provides better angular resolution than many other multiple-fibre reconstruction algorithms, while maintaining a modest computation time (Tournier *et al.*, 2007; Seunarine and Alexander, 2013). The CSD reconstruction used a maximum spherical harmonic order of 6 for both the response and the fibre orientation distribution functions. A summary of the processing pipeline is provided in Fig. 1.

### Diffusion tractography

Whole brain probabilistic tractography was performed using the iFOD2 algorithm in MRtrix (Tournier *et al.*, 2012). Specifically, 5 million streamlines were seeded throughout the white matter, in all foreground voxels where fractional anisotropy <0.2. Streamlines were terminated when they either reached the cortical or subcortical grey matter mask or exited the foreground mask. The SIFT algorithm (spherical-deconvolution informed filtering of tractograms; Smith *et al.*, 2013) was then used to reduce biases in the reconstructed data by ensuring that the streamline densities were proportional to the estimated fibre density. The resulting set of streamlines was used to construct the structural brain network.

For the corticobasal ganglia connectivity analysis (including VCPs), 5000 streamlines were seeded for each voxel within the basal ganglia regions of interest and terminated when they reached the cortical mask or exited the hemisphere mask. The probability of connectivity between every seed voxel and every target region was established for each subject and the data were stored as individual subject connectivity probability maps.

#### Construction of structural brain network

Regions of interest were defined as connected if a fibre originated in Region of interest 1 and terminated in Region of interest 2. These connections were weighted by streamline count and combined into a 76 × 76, undirected and weighted, structural connectivity matrix. In addition we also created volume normalized matrices for our analyses. This was done by dividing the number of streamlines connecting two regions of interest by the sum of their volumes. Both un-normalized and normalized matrices were used in the analysis as it is unclear from the current literature whether normalization is required or not.

The threshold masks for generating sparse connectivity matrices for the subsequent graph theoretic analyses were created for connections present in 25%, 50%, 75% and 100% of control participants. These masks were then used to threshold individual connectivity matrices across all groups, consistent with thresholding strategies in the seminal rich club paper (van den Heuvel and Sporns, 2011). By using the control group to generate threshold masks we aimed to exclude connections due to noise as opposed to pathology. Whole brain network results were most consistent across thresholds for un-normalized matrices (Supplementary Table 2) therefore un-normalized results are reported here, whereas normalized are reported in Supplementary Tables 2 and 3. Both un-normalized and normalized results are generally in agreement unless otherwise stated in the manuscript. As results were consistent across thresholds a value of 75% was used, in keeping with the chosen threshold in the seminal human rich club report (van den Heuvel and Sporns, 2011).

### Graph theoretical analysis

Various graph metrics were calculated using the brain connectivity toolbox (Rubinov and Sporns, 2010) and have been discussed in detail elsewhere (Bullmore and Sporns, 2009). We analysed the structural networks using both global and local nodal summary statistics. Global graph metrics characterize the brain network properties as a whole whereas using node-level local metrics we can probe more region-based differences.

Global brain network segregation was assessed using normalized clustering coefficient and modularity. Clustering coefficient is the fraction of brain regions neighbours that are also neighbours of each other. Modularity represents the community structure present within brain networks. Brain network integration was assessed using normalized average path length and global efficiency. Average path length represents the average of shortest paths between brain regions in the network; increase in average path length represents loss of network integration. Global efficiency is the inverse of shortest path length. Small worldness was also investigated. Networks that exhibit small worldness show a fine balance between network integration and segregation to facilitate both regional and network-wide information processing. Path lengths and clustering coefficients were normalized relative to a set of 1000 random networks.

Altered topology in individual brain regions was assessed using degree, strength, betweenness centrality and clustering coefficients. Degree is defined as the number of connections to a brain region that link it to the rest of the network whereas strength is the weighted variant of degree. Betweenness centrality is defined as the fraction of shortest paths in the network that pass through a given brain region. For ease of understanding, betweenness centrality is referred to as ‘network traffic’ throughout the manuscript. Similarly, strength is referred to as (graph theory) strength to avoid confusion with streamline density.

Statistically significant group differences in graph metrics were analysed using permutation testing (10 000 permutations) with two-tailed *t*-tests to investigate both increases and decreases in structural connectivity. Age, sex, education and study site were included as covariates. For individual brain region metrics, a false discovery rate (FDR) correction was applied across the 76 brain regions, and a Bonferroni correction was applied for the multiple graph theory measures tested in both the regional and whole brain analyses.

### Rich club analysis

A rich club analysis (van den Heuvel and Sporns, 2011) was performed to identify rich club organization and regions in each group. Rich club organization is a tendency for highly connected brain regions to be more densely connected among themselves than brain regions with fewer connections. The weighted rich club coefficients were calculated for each participant and normalized relative to a set of 1000 comparable random networks. The presence of rich club organization was identified by performing two-tailed *t*-test permutation testing (10 000 permutations) of the area under the curve (normalized weighted rich club coefficient against degree) for each group versus random network. FDR correction was then applied to correct for multiple comparisons across a range of degrees, in line with a similar analysis in the literature (van den Heuvel *et al.*, 2013). Rich club regions were defined as the top 12 brain regions with the highest degree. To ensure reliability of our tractography data, rich club regions were also investigated in the 80-region analysis for combined and single sites (Supplementary material).

#### Clinical correlations

We investigated how differences in brain networks relate to performance in cognitive and motor tests. Participants were assessed using symbol digit modalities (Smith, 1968), Stroop word reading (Stroop, 1935), Indirect Circle Tracing (log of the indirect circle annulus) (Say *et al.*, 2011; Tabrizi *et al.*, 2011), Negative Emotion Recognition (Ekman and Friesen, 1976), Speeded Tapping (mean inter-tap interval non-dominant hand) (Reilmann, 2005) and total motor score (Huntington Study Group, 1996). These variables were chosen because of their demonstrated sensitivity in Huntington’s disease (Stout *et al.*, 2012; Tabrizi *et al.*, 2013). We used partial Pearson correlations controlling for age, sex, study site, education and CAG length to assess how graph metrics related to clinical variables for Huntington’s disease gene carriers (additionally correlations for graph metrics of individual groups for the 80-region analysis are presented in the supplementary material). Regional correlations were FDR corrected across 76 brain regions and Bonferroni corrected for four local metrics and six clinical measures (*P* < 0.05/24). Whole brain correlations were Bonferroni corrected for five global metrics.

### Network-based statistics

To probe further which specific structural connections showed group differences in premanifest Huntington’s disease versus controls we used the network-based statistics method (Zalesky *et al.*, 2010). A general linear model was used to model group differences with age, sex, education and study site included as covariates. Permutation testing, using unpaired *t-*tests, was performed with 5000 permutations. A test statistic was then computed for each connection and a threshold applied (*t* = 3.1) to produce a set of suprathreshold connections, thereby identifying anatomical networks, which show significant differences in structural connectivity between groups. A family-wise error correction was also applied (*P* < 0.05).

### Selective vulnerability: streamline density, network traffic, regional clustering and distance from the striatum

Correlations were performed between the average control group streamline density for each brain connection against group differences in streamline density for that brain connection, both for all brain connections in the network and for corticobasal ganglia connections only. For corticobasal ganglia connections only, separate analyses were performed using streamline data generated from the graph theory pipeline (volume un-normalized and normalized) and from the corticobasal ganglia connectivity pipeline (volume normalized only). Average control brain region network traffic was correlated with group differences in (graph theory) strength and degree for each brain region. We also performed correlations between regional clustering coefficient and group differences between degree and (graph theory) strength for each brain region. Partial Pearson correlations, controlling for Euclidean distance, were performed for average control path length to the basal ganglia and group differences in connection density for each corticobasal ganglia connection, separate analyses were performed using streamline data generated from the graph theory pipeline (volume un-normalized and normalized) and from the corticobasal ganglia connectivity pipeline (volume normalized only). For this analysis, a Bonferroni correction was applied for (27) multiple comparisons.

### Group-averaged voxel connectivity profiles

Group-averaged VCPs were generated to allow qualitative analysis of ‘patterns’ of basal ganglia connectivity. To generate group-averaged VCPs, the individual connectivity probability maps (defined in the ‘Diffusion Tractography’ section above) were first warped into standard space using Niftyreg. Specifically, we used a two-step approach, warping the connectivity maps to their corresponding subject T_1_-weighted image space, before warping the resulting image into MNI space. The standard space connectivity maps were then averaged together to create a mean connectivity map for each group. VCPs were generated using the approach described by Draganski *et al.* (2008). Finally, the labels of the VCPs were standardized for each structure (i.e. so that the labels for the structure are comparable between groups) by finding the unique patterns of connectivity across all three groups, assigning a new label to each pattern in the set and then remapping the labels of the VCPs to the new scheme.

### Statistical analysis of corticobasal ganglia connectivity

Statistical analysis of the corticobasal ganglia connectivity information was performed on the individual connectivity maps using a mass-univariate approach. This involved permutation testing as outlined in the graph theoretical analysis. Age, gender, study site and education were included as covariates when assessing group differences. CAG length was also included as a covariate in the correlation analyses. An FDR correction was applied for 210 corticobasal ganglia connections and a Bonferroni correction applied for six clinical tests. The inputs to both analyses were vectors describing the volume of the basal ganglia regions of interest that connected to each of the cortical targets. Specifically, the individual connectivity maps were first binarized such that any voxel within the basal ganglia region of interest with at least 1% of streamlines reaching a given cortical target was regarded as being connected to that target. The number of voxels connected to the cortical target were then calculated and normalized by the sum of the volumes of the corresponding basal ganglia region of interest and cortical target, providing a normalized estimate of the volume of the region connected to target. The procedure was repeated for all cortical targets, resulting in a vector describing the connectivity between the basal ganglia and cortex for each subject.

## Results

### Cohort

The cohort consisted of 38 participants with early Huntington’s disease (25 female; mean age 49.5 ± 10.4 years; mean CAG repeat length 43.4 ± 2.4, mean disease burden score 370.6 ± 10.4), 50 premanifest Huntington’s disease participants (24 female; mean age 42.2 ± 8.9 years; mean CAG repeat length 40.3 ± 2.1, estimated years to onset 11.1 ± 3.9, mean disease burden score 301.3 ± 7.4) and 47 controls (32 female; mean age 47.6 ± 9.0 years). Premanifest Huntington’s disease participants were significantly younger in age when compared with controls (*P* = 0.006) and Huntington’s disease (*P* = 0.0005) participants. There were no group differences in gender, education or study site (Supplementary Table 1). The analysis was pseudo-longitudinal in that we included two different stages of the same condition with premanifest and manifest Huntington’s disease having a temporal spacing of at least 10 years (Langbehn *et al.*, 2004).

### Rich club organization

All groups showed significant rich club organization (Control; degree 20–63, Premanifest; degree 19–63, Manifest; degree 19–63). Rich club regions were in perfect agreement across groups. The regions were as follows (in order of highest degree): right and left thalamus, precuneus and caudate, right superior parietal, left superior frontal, right superior frontal, left superior parietal, right insula, left insula. The same rich club regions were identified for the 80-region analysis both at combined and single sites (Supplementary material).

### Regional brain network measures

**Figure 2:**
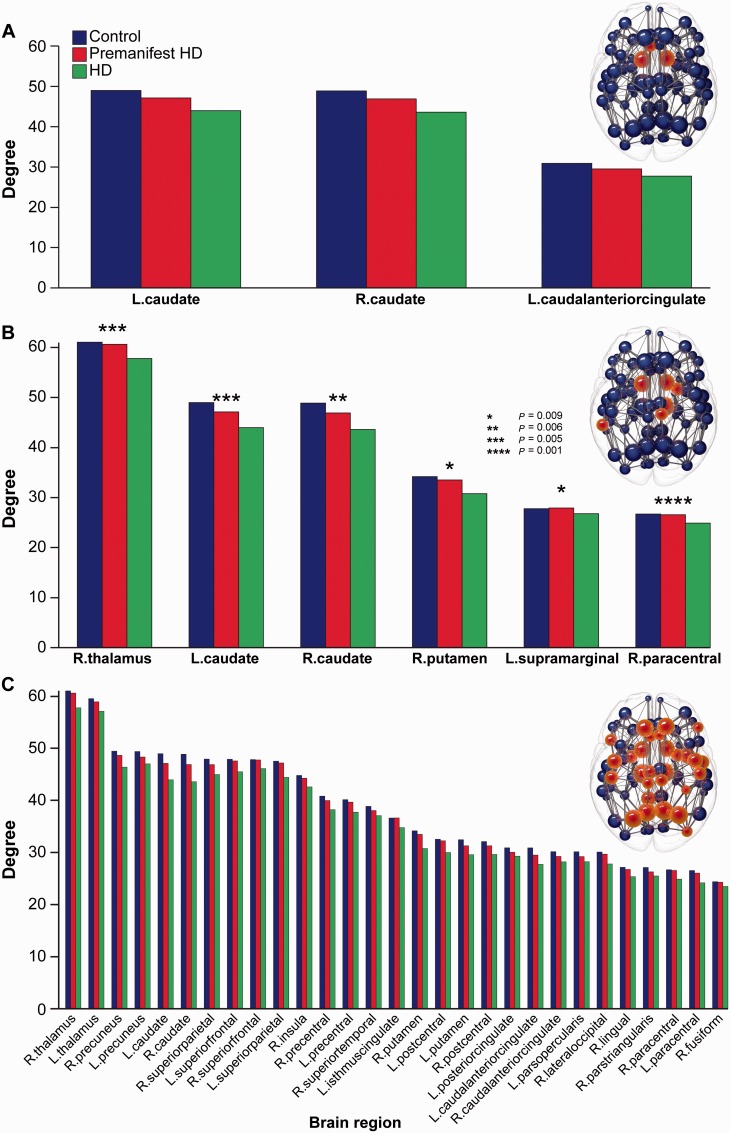
**Group differences in degree**. Significant group differences in degree for (**A**) premanifest Huntington’s disease versus controls (*P = *0.01 for all regions), (**B**) Huntington’s versus premanifest Huntington’s disease (*P = 0.009, **P = 0.006, ***P = 0.005, ****P = 0.001) and (**C**) Huntington’s disease versus controls. For **C,** only those regions with *P* < 0.0003 are displayed to highlight the most significant regions. Controls (blue, left columns), premanifest Huntington's disease (red, centre columns) and Huntington's disease (green right columns) are presented in each graph to illustrate consistent step-wise reductions in degree across groups. A brain network is displayed above each bar chart. Spheres represent brain regions with red spheres indicating the brain regions showing significance between groups. Data are represented as a group mean (confidence intervals are not included as not standard for permutation tests).

Significant reductions in degree (number of brain connections) were seen between premanifest Huntington’s disease and controls in the left and right caudate and left anterior cingulate (Fig. 2A). Manifest versus premanifest Huntington’s disease showed significant reductions in the left and right caudate, right thalamus, right putamen, right paracentral and left supramarginal regions (Fig. 2B). Numerous regions showed significant decreases in Huntington’s disease versus controls including cortical and basal ganglia rich club regions, as well as cingulate, motor, temporal and occipital areas (Fig. 2C). Volume normalized results and the three additional graph metrics examined [strength, betweenness centrality (network traffic) and clustering coefficient] are presented in Supplementary Table 3. Similar group differences were seen in the 80-region analysis (Supplementary Fig. 1) with the exception of (graph theory) strength (Supplementary material).

### Network segregation

**Figure 3:**
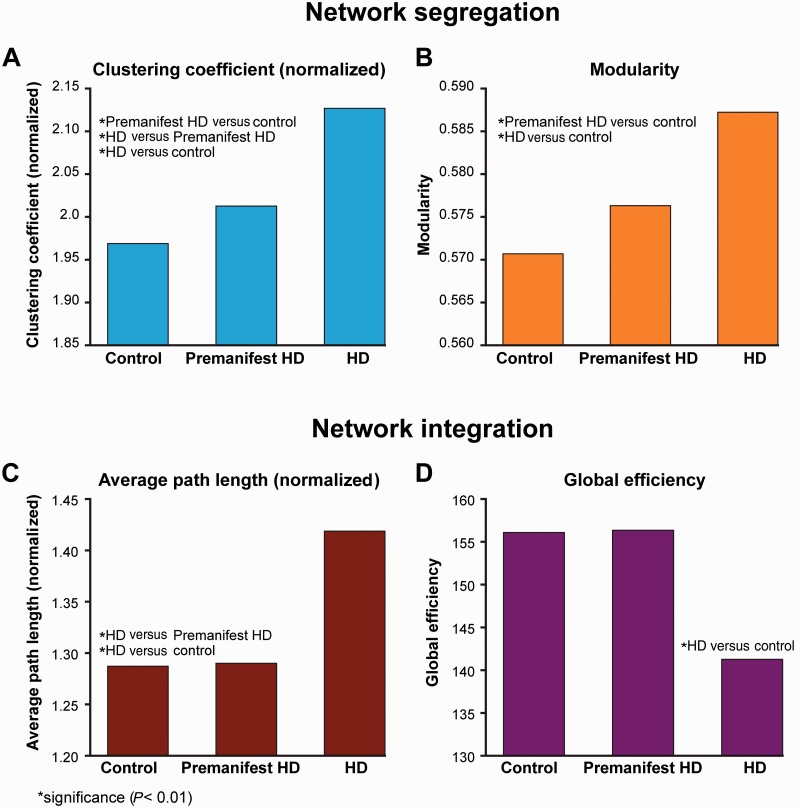
**Group differences in network segregation and integration**. Segregation: (**A**) normalized clustering coefficient (**B**) modularity. Integration: (**C**) normalized average path length and (**D**) global efficiency. *P < 0.01. Data are represented as a group mean (confidence intervals are not included as not standard for permutation tests). HD = Huntington’s disease.

Significant increases were seen in normalized clustering coefficient in Huntington’s disease participants versus controls (*P* = 0.0001), Huntington’s disease participants versus premanifest (*P* = 0.0007) and premanifest participants versus controls (*P* = 0.0082) (Fig. 3A). Modularity showed significant increases in Huntington’s disease participants versus controls (*P* = 0.0001) and in premanifest Huntington’s disease participants versus controls (*P* =0.0037) (Fig. 3B). Similar group differences in segregation were seen in the 80-region analysis (Supplementary material)

### Network integration

Normalized average path length showed significant increases in Huntington’s disease participants versus controls (*P* = 0.0004) and manifest versus premanifest Huntington’s disease participants (*P* = 0.0032) (Fig. 3C). Significant decreases were seen in global efficiency in Huntington’s disease participants versus controls (*P* = 0.006) (Fig. 3D). No significant group differences were seen in small worldness. The 80-region analysis revealed similar group differences in network integration (Supplementary material).

### Corticobasal ganglia connections

**Figure 4:**
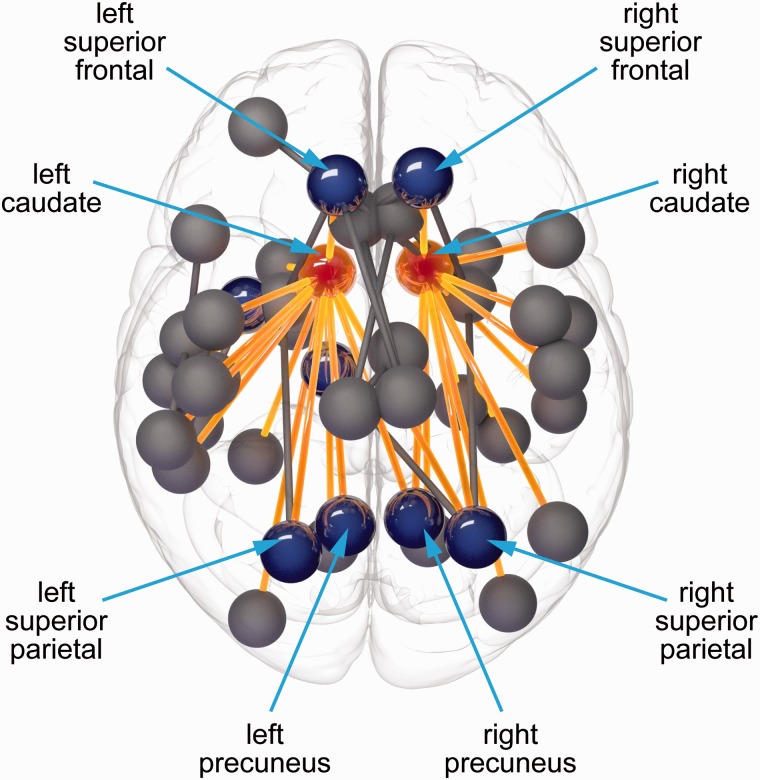
**Network-based statistics analysis showing significantly reduced connectivity between premanifest Huntington’s disease versus controls in cortico-caudate connections**. Red = caudate; blue = cortical rich club regions; yellow = cortico-caudate connections.

When comparing premanifest Huntington’s disease participants versus control subjects significant reductions were predominantly seen in cortico-caudate connections in the network-based statistics analysis (Fig. 4). These included a number of basal ganglia connections to cortical rich club and non-rich club regions (Supplementary Table 4).

**Figure 5:**
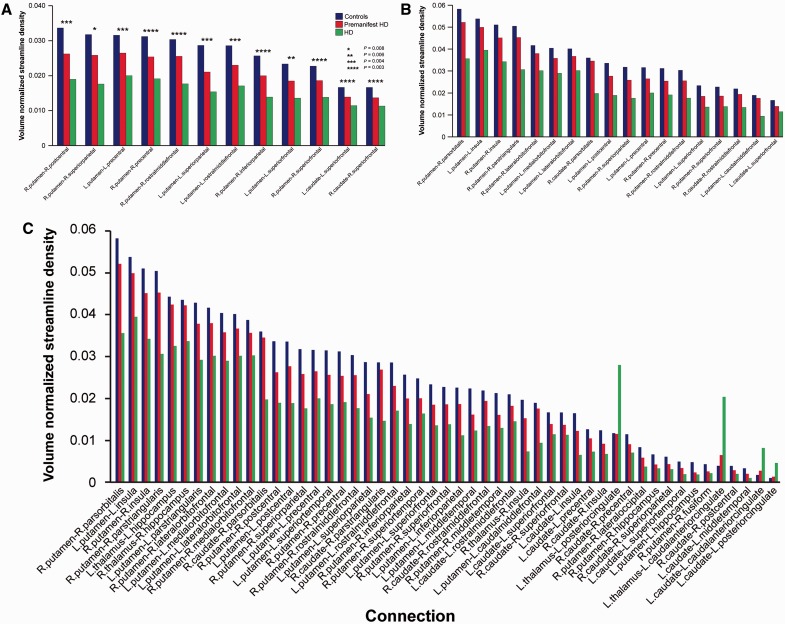
**Corticobasal ganglia connectivity univariate analysis**. Group differences between (**A**) premanifest Huntington’s disease (HD) versus controls (*P = 0.008, **P = 0.006, ***P = 0.004, ****P = 0.003), (**B**) Huntington’s disease versus premanifest Huntington’s disease, and (**C**) Huntington’s disease versus controls. Only those connections with (**A**) *P* < 0.009, (**B**) and (**C**) *P* < 0.002 are displayed to highlight most significant connections. Data are represented as a group mean (confidence intervals are not included as not standard for permutation tests). Controls are shown in blue, left columns; premanifest Huntington’s disease is shown in red, centre columns; and Huntington’s disease is shown in green, right columns.

The corticobasal ganglia connectivity univariate analysis (*P* < 0.05), using streamline density, showed that of those connections showing group differences, 57% (13/23) connected to rich club regions for premanifest Huntington’s disease participants versus controls, 51% (18/35) for manifest versus premanifest Huntington’s disease participants and 68% (75/111) for Huntington’s disease participants versus controls (Fig. 5; see Supplementary Fig. 3 for landscape version of Fig. 5C). For Huntington’s disease participants versus controls increases were seen in streamline density in connections to the anterior and posterior cingulate

### Altered patterns of corticobasal ganglia connectivity

**Figure 6:**
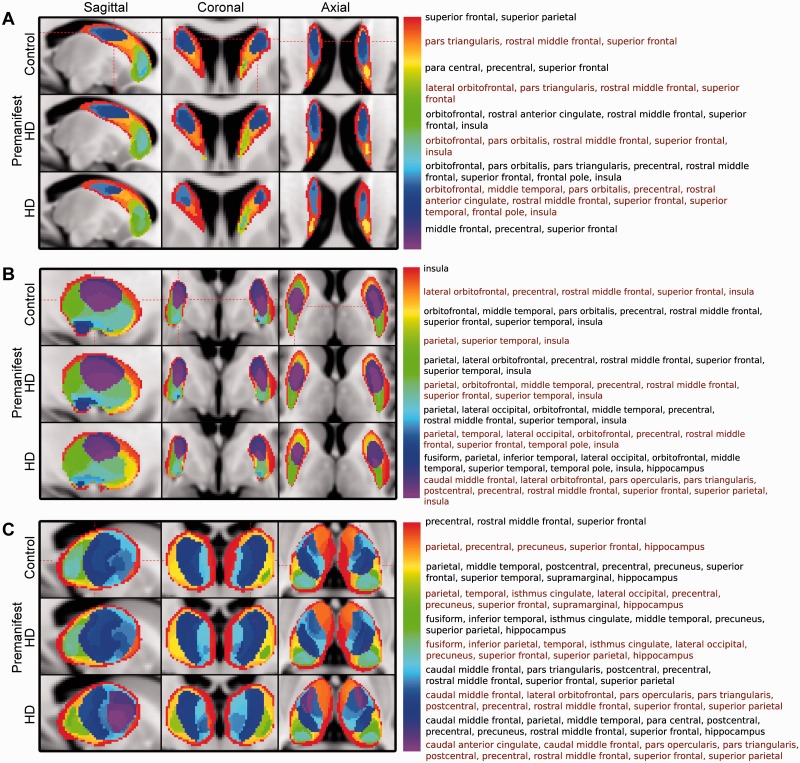
**VCP across groups.** (**A**) Caudate, (**B**) putamen, and (**C**) thalamus. Labels are combined as follows: parietal = superior and inferior, orbitofrontal = lateral and medial, temporal = inferior, middle and superior. Each label list corresponds to the colour in the legend to the left of it. Label lists are displayed in black and red font alternately for ease of viewing.

Group averaged VCPs showed altered patterns of cortical connectivity to the caudate, putamen and thalamus, with all connectivity patterns including one or more cortical rich club regions. In the caudate there was loss of connectivity to the superior frontal and insula rich club regions in manifest participants compared with controls and premanifest Huntington’s disease (Fig. 6A). The putamen shows loss of connectivity to the superior frontal, superior parietal and insula rich club regions both in manifest and premanifest Huntington’s disease compared to controls (Fig. 6B), whereas the thalamus showed loss of connectivity to the superior parietal, precuneus and superior frontal rich club regions in manifest compared to premanifest Huntington’s disease and controls (Fig. 6C).

### Regional brain network clinical correlations

Clinical measures revealed correlations with the degree (number of brain connections) of rich club and non-rich club brain regions (DF = 86, for all clinical correlations). For total motor score, correlations were seen with the left (Rho = −0.48, *P = *2.6 × 10^−4^) and right inferior parietal (Rho = −0.44, *P = *0.001), left caudal middle frontal (Rho = −0.43, *P = *0.001), left precentral (Rho = −0.4, *P = *0.002), left superior frontal (Rho = −0.41, *P = *0.002) and left rostral middle frontal regions (Rho = −0.4, *P = *0.002). Indirect circle tracing correlated with the left superior frontal (Rho = 0.45, *P = *0.002) and right lingual (Rho = 0.43, *P = *0.002), while Negative Emotion Recognition test performance correlated with the right caudate (Rho = 0.49, *P = *2.3 × 10^−4^), left inferior parietal (Rho = 0.44, *P = *0.001) and left precentral (Rho = 0.43, *P = *0.001).

No significant (*P* < 0.05) correlations were seen with any other regional graph metric and Stroop word reading, symbol digit modalities or Speeded Tapping mean inter-tap interval. Similar correlations were seen in the 80-region analysis for gene carriers only and across groups (Supplementary material and Supplementary Fig. 2).

### Whole brain network clinical correlations

Network segregation: normalized clustering coefficient showed significant correlations with total motor score (Rho = 0.42, *P = *7.55 × 10^−5^) and indirect circle tracing (Rho − 0.43, *P = *7.62 × 10^−5^), while modularity significantly correlated with total motor score (Rho = 0.34, *P = *1.15 × 10^−6^). Network integration: correlations were seen for normalized average path length and total motor score (Rho = 0.51, *P = *1.15 × 10^−6^) and emotion recognition (Rho = −0.34, *P = *0.002). The 80-region analysis revealed similar correlations (Supplementary material).

### Clinical correlation with corticobasal ganglia connectivity

For the corticobasal ganglia connectivity analysis our univariate results showed significant negative correlations with total motor score and streamline density for 27 corticobasal ganglia connections, 16 (59%) of which were connected to rich club regions.

### Susceptibility to structural connectivity loss

**Figure 7:**
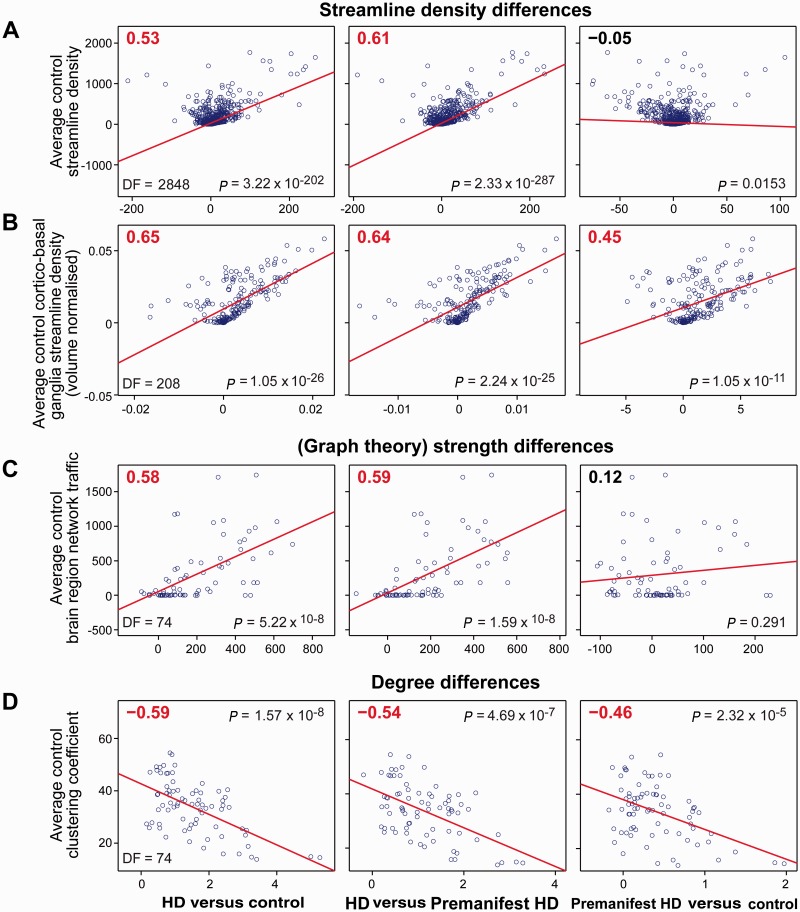
**Selective vulnerability analysis**. (**A**) Correlation of average control streamline density against group differences in streamline density [based on graph theoretical analysis (volume un-normalized)]; each data point represents a single brain connection. (**B**) Correlation of average control corticobasal ganglia streamline density against group differences in streamline density [based on corticobasal ganglia connectivity analysis (volume normalized)]; each data point represents a single corticobasal ganglia connection. (**C**) Correlation of average control brain region network traffic against group differences in (graph theory) strength [based on graph theoretical analysis (volume un-normalized)]; each data point represents a single region of interest. (**D**) Correlation of average control clustering coefficient against group differences in degree [based on graph theoretical analysis (volume un-normalized)]; each data point represents a single region of interest. HD = Huntington’s disease.

Significant (*P* < 0.05 corrected) positive correlations were identified between average control streamline density and group differences in streamline density for Huntington’s disease versus controls. Group differences in streamline density in premanifest versus control subjects did not show a correlation (Fig. 7A). Similar results were seen for corticobasal ganglia connections in both the graph theory and corticobasal ganglia connectivity analysis. A positive correlation was seen between average control streamline density and group differences in streamline density for premanifest versus controls in the corticobasal ganglia connectivity analysis (Fig. 7B).

Significant positive correlations were seen with average control brain region network traffic and group differences in (graph theory) strength across all groups (Fig. 7C); however, this was not maintained after volume normalization (Supplementary Table 5). No significant correlations were seen with average control brain region network traffic and group differences in degree.

Significant negative correlations were seen with average control clustering coefficient and group differences in degree across all groups (Fig. 7D). No significant correlations were seen with average control clustering coefficient and group differences in (graph theory) strength.

There were no significant correlations between path length to the basal ganglia and group differences in streamline density for either the graph theory or corticobasal ganglia connectivity analysis. Volume normalized results are provided in Supplementary Table 5.

## Discussion

### Altered brain network connectivity and selective vulnerability in premanifest and manifest Huntington’s disease

**Figure 8:**
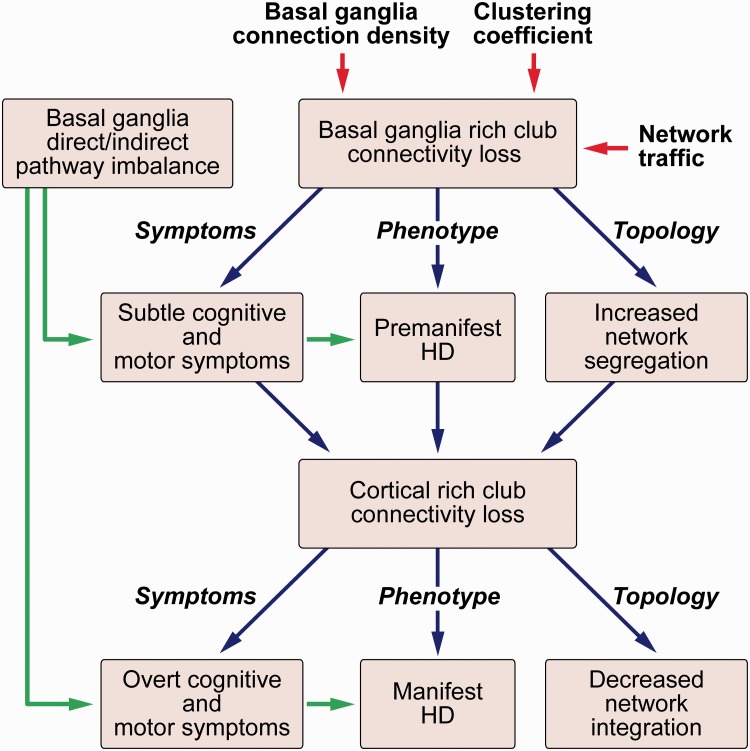
**Summary of findings**. There is selective loss of basal ganglia rich club connectivity due to high higher connection to the basal ganglia, higher network traffic and reduced clustering coefficients of rich club regions. This results in increased network segregation leading to the subtle motor and cognitive symptoms seen in premanifest Huntington’s disease. Further loss of cortical rich club connectivity results in reduced network integration resulting in the overt cognitive and motor symptoms seen in manifest Huntington’s disease. HD = Huntington’s disease.

We performed a graph theory analysis, complemented by network-based statistics, corticobasal ganglia connectivity and VCP analyses to focus on structural connectivity loss of rich club regions in Huntington’s disease. We found altered brain network connectivity specifically affecting rich club regions, predominantly in the basal ganglia (caudate) in premanifest Huntington’s disease, but extending to cortical rich club regions (superior frontal, superior parietal, precuneus and insula) in manifest disease. By using network-based statistics, corticobasal ganglia connectivity and VCP analyses, we were also able to demonstrate selective loss of connections and altered patterns of connectivity between the basal ganglia and cortical rich club regions. In conjunction with these regional group differences we identified altered whole brain network topology with isolated increase of network segregation in the premanifest Huntington’s disease participants when compared to controls; this extended to an increase in segregation and loss of integration when comparing manifest against both premanifest Huntington’s disease and controls. This suggests that increases of whole brain network segregation occur in the earliest stages of the neurodegenerative disease process, before symptom onset, and subsequently progress to loss of network integration in the manifest stages. We postulate that conversion from premanifest to manifest Huntington’s disease, in addition to the emergence of chorea through an imbalance in the indirect and direct pathways of the basal ganglia (Andre *et al.*, 2011), may reflect a breakdown of such network integration (Fig. 8).

While reduction in the degree of rich club brain regions was seen at a regional level, increase in network segregation was seen at a global level in both premanifest and manifest Huntington’s disease. Connections among rich club brain regions make up the majority of long-range connections in the human connectome (van den Heuvel *et al.*, 2012). This suggests that the loss of these long-range rich club connections connecting distant brain regions results in increased network segregation in the premanifest stage, and loss of integration in the manifest stage. In contrast to degree of the rich club regions, relatively few group differences were seen in network traffic (betweenness centrality), (graph theory) strength and clustering coefficient. Network traffic is the most sensitive graph theory measure in traumatic brain injury (Fagerholm *et al.*, 2015). However, our data show that Huntington’s disease predominantly results in loss of brain connections (degree) as opposed to alterations of regional brain network topography.

Rich club regions are brain hubs that form the backbone of the brain network (van den Heuvel *et al.*, 2012) allowing integration of specialized cortical regions (Senden *et al.*, 2014). We demonstrate positive correlation with emotion recognition performance and degree of the right caudate and left superior frontal region. It is unsurprising that loss of degree to these rich club regions is associated with impaired emotion recognition. Previous work by our group, using task functional MRI in premanifest Huntington’s disease, showed a number of the regions defined as rich club (for example caudate, thalamus, superior frontal, superior parietal, precuneus and insula) are activated when performing an emotion recognition task (Novak *et al.*, 2012). We also report negative correlation between emotion recognition performance and normalized clustering coefficient (a measure of network segregation). This suggests increased segregation of the network leads to impaired communication between specialized brain regions resulting in impaired emotion recognition performance. Similar results are seen when we correlate total motor score with degree of the left superior frontal, normalized clustering coefficient and normalized average path length (a measure of network integration). Thus loss of rich club connectivity, coupled with increased network segregation and reduced integration is likely to contribute to the clinical manifestation of Huntington’s disease. The lack of correlation of Stroop word reading, symbol digit modalities or Speeded Tapping mean inter-tap interval with rich club graph theory measures suggest that these tasks may be less dependent on optimal communication between diverse brain regions.

By testing several mechanistic hypotheses, we shed light on the reasons for the region and connection selective changes we have shown in premanifest and manifest Huntington’s disease. We demonstrated a strong positive correlation with streamline density (particularly to the basal ganglia) and group differences in streamline density. We show correlations between group differences in (graph theory) strength and brain region network traffic. Furthermore we show negative correlation between regional clustering coefficient and group differences in degree. In contrast to a previous study in Alzheimer’s disease and frontotemporal dementia (Zhou *et al.*, 2012), we found a very low correlation with group differences in streamline density and path length to the area showing earliest atrophy, which in Huntington’s disease is the striatum (Tabrizi *et al.*, 2011). The reason for this difference is likely methodological. Zhou and colleagues (2012) based their calculation of path length on resting state functional MRI data of healthy controls and correlated path length with voxel based morphometry atrophy patterns seen in dementia patterns. In contrast, we used diffusion tractography to generate both variables to examine correlation between path length to the striatum and group difference in streamline density, which we suggest may be a more robust approach.

In other neurodegenerative disorders, similar whole-brain network disruption is seen. In Alzheimer’s disease there is a loss of both network integration and segregation in the structural connectome, which is also seen to a lesser extent in mild cognitive impairment (Yao *et al.*, 2010; Bai *et al.*, 2012). Mild cognitive impairment is a syndrome with increased risk of developing Alzheimer’s disease, which can perhaps be thought of as loosely comparable to premanifest Huntington’s disease, in that it is a preclinical form of Alzheimer’s disease. However, studies of premanifest Huntington’s disease are more specific, in that 100% of premanifest Huntington’s disease gene carriers will develop the disease, whereas only a much smaller proportion of participants with mild cognitive impairment develop Alzheimer’s disease. While the authors of a recent review suggest increased network segregation is a direct consequence of loss of integration (Griffa *et al.*, 2013), our results suggest that these network phenomena are not directly linked but rather develop ‘sequentially’ as the disease progresses.

The findings of this study have direct relevance for many of the emerging therapeutic strategies in Huntington’s disease. The conceptually most compelling therapeutic strategy is gene silencing using a range of potential agents/compounds (Godinho *et al.*, 2015). However one challenge to the potential effectiveness of such therapies is their ability to distribute widely enough and to reach therapeutically relevant concentrations in the brain (Wild and Tabrizi, 2014). A recent animal study suggests that when mutant HTT is still present in the striatum, but removed from the cerebral cortex, there is improvement in motor and behavioural deficits (Wang *et al.*, 2014). This is consistent with our results showing that there is preserved global efficiency when mainly cortico-striatal rich club connections are affected, in the premanifest stage, when gene carriers have subtle symptoms. It may therefore be the case that if antisense oligonucleotides are only able to target the cortex this may cause sufficient huntingtin lowering to preserve structural network integrity and prevent conversion to manifest Huntington’s disease.

Our study is the first to investigate the structural connectome in Huntington’s disease using graph theory. Previously, Poudel *et al.* (2014) used deterministic tractography and network-based statistics to investigate fractional anisotropy, radial diffusivity and streamline density changes in a frontal-parietal-striatal network in premanifest and manifest Huntington’s disease and control participants. They showed group differences of these metrics in the frontal-parietal-striatal network and correlations of these metrics with cognitive and motor variables. However, the use of deterministic tractography with the diffusion tensor formalism is incapable of resolving crossing fibres (Behrens *et al.*, 2007). Furthermore the study is limited to a network-based statistical analysis of the frontal-parietal-striatal network and was therefore unable to address the selective vulnerability of rich club brain regions or global changes in the brain network as we have done in this work.

For the first time in a diseased population, we used both CSD, which deals more effectively with crossing fibres than the diffusion tensor or multi-tensor methods (Tournier *et al.*, 2012) and SIFT, which has higher reproducibility and is more representative of the underlying biology of white matter connections than conventional methods (Smith *et al.*, 2015). CSD has been shown to perform well at the acquisition protocol specifications used in this study (b = 1000) (Ramirez-Manzanares *et al.*, 2011; Wilkins *et al.*, 2015). At b = 1000 a minimum number of 28 gradient directions is required (Tournier *et al.*, 2013). Therefore the angular coverage achieved using CSD at b = 1000 is more than sufficient with 42 directions. While the performance of diffusion tractography methods in patients with atrophy has not been explicitly examined in the literature, previous work by our group has demonstrated low within-subject variability of diffusion metrics in manifest Huntington’s disease participants, suggesting atrophy does not cause significant distortion of the diffusion signal (Cole *et al.*, 2014).

We seed streamlines using two complimentary approaches. In the graph theory analysis streamlines are seeded throughout the white matter and allowed to connect to any of our 76 regions of interest. In contrast, for the corticobasal ganglia analysis, streamlines are seeded in the grey matter of basal ganglia structures at the voxel level and allowed to connect to multiple regions in their corresponding hemisphere. This allows us to probe corticobasal ganglia structural connectivity change both at the region of interest and voxel level, therefore taking into account local basal ganglia changes in architecture that one may expect in Huntington’s disease.

We used this latter approach in combination with canonical variate analysis, a multivariate technique, and VCP analyses in a previous study where we failed to find group differences between premanifest Huntington’s disease and controls (Novak *et al.*, 2015). This was likely due to methodological differences, particularly the use of multitensor reconstruction as opposed to CSD used in this study. While the previous study suggested a relative increase in structural connectivity to the putamen, volume normalization was only carried out on basal ganglia structures. In this study we volume normalized for all brain regions. Furthermore, because canonical variate analysis-based multivariate analysis has limited interpretation in terms of suggesting changes in individual connections’ contribution to the overall connectivity pattern; in this study we performed a univariate analysis to uncover direction of change in connectivity. This univariate analysis did not suggest any increase in connectivity *per se* as was reported in Novak *et al.* (2015), based on canonical variate analysis analysis.

Our results suggest that loss of subcortical rich club connectivity in the premanifest stage of Huntington’s disease leads to increased network segregation followed by loss of cortical rich club connectivity after clinical disease manifests causing a breakdown of network integration. This study was, however, pseudo-longitudinal, and thus further longitudinal analysis is required to confirm the progression in structural brain network change over time in Huntington’s disease. However, studying adult onset slowly progressive neurodegenerative diseases longitudinally is challenging as the participants will need to be followed for decades or longer, particularly in those with premanifest disease. Pseudo-longitudinal analyses allow a snapshot that encompasses, in one analysis, a timespan of over 25 years with premanifest Huntington’s disease gene carriers up to 15 years before predicted onset (Langbehn *et al.*, 2004) and early stage clinically symptomatic participants (up to 10 years after onset).

With respect to limitations, there were significant differences in age between premanifest and control subjects and participants with premanifest and manifest Huntington’s disease in our cohort, an unavoidable consequence of the natural history of Huntington’s disease. We aimed to minimize this effect by including age as a covariate of no interest in all analyses to model and remove this variance. We acknowledge that streamline density is not a direct marker of axonal fibre count. We also acknowledge the limitations of diffusion tractography. However, we have taken steps to address the biases that exist in diffusion tractography, namely longer streamlines are more likely to terminate prematurely whereas regions of interest closer together have shorter streamlines and are therefore likely to have higher ‘connection densities’. To overcome these limitations we used the SIFT method which is more reproducible and biologically accurate than conventional methods (Smith *et al.*, 2015).

This study focuses on interbrain region structural connectivity. Therefore abnormalities occurring within specific brain regions, such as the caudate and putamen, have not been taken into account. We do, however, account for brain region atrophy by reporting both volume un-normalized and volume normalized results.

Currently in the literature there is no consensus regarding volume normalization in connectome studies. There is a suggestion that volume normalization may overcompensate volume-driven effects on streamline count (Zalesky and Fornito, 2009). We have found results suggestive of this in our study. In our graph theoretical analysis, volume normalized results show increases in strength in the left thalamus and left hippocampus in Huntington’s disease versus controls. Additionally in the corticobasal ganglia connectivity analysis increases were seen in connection density in connections to the anterior and posterior cingulate. While there is a suggestion that compensatory mechanisms come into play in Huntington’s disease, these are more likely to occur in the premanifest stage (Papoutsi *et al.*, 2014; Scheller *et al.*, 2014). This suggests that our results showing increased (graph theory) strength or connection densities in these regions are spurious results of volume normalization. Similarly, in assessing regional betweenness centrality and group differences in (graph theory) strength, the positive correlation we observed in the un-normalized results was not maintained after volume normalization. While the optimal choice of brain parcellation scheme in connectome studies is unknown, some authors suggest that less dense parcellation schemes with larger regions of interest allow for more robust reproducible findings than dense parcellation schemes with thousands of regions of interest (Smith *et al.*, 2015).

## Conclusion

We show highly connected brain regions, with high network traffic, are most susceptible to structural connectivity loss in Huntington’s disease, which results in clinically relevant brain network changes of increased segregation in the premanifest stage and loss of integration in the manifest stage. These findings highlight the role of the rich club as a substrate for the structural connectivity loss seen in Huntington’s disease and have broader implications for understanding the connection between molecular and systems level pathology in neurodegenerative disease.

## Supplementary Material

Supplementary Table 1

## Abbreviations

CSD: constrained spherical deconvolution
VCP: voxel connectivity profile

## Appendix 1

### TRACK-HD Investigators

Stefan Bohlen, Muenster;Helen Crawford, UCL; Ellen P Hart, LUMC, Leiden; Nicola Z. Hobbs, UCL; Celine Jauffret, Paris; Hélène Francisque, Paris; Izelle Labuschagne, Monash; Nayana Lahiri, UCL; Stéphane Lehericy, Paris; Marie Laure Monin, Paris; Ian Malone, UCL; Alison O’Regan, Monash; Sarah Queller, Indiana; Joy Read, UCL; Ralf Reilmann, Meunster/GHI; Verena Rödig, LUMC, Leiden; Romain Valabrègue, Paris; Jeroen van der Grond, LUMC, Leiden; Daisy Whitehead, UCL; Felix Ttita, Ulm; Katja Vitkin, Ulm; Theresia Kelm, Ulm; Rosine Scherer Ulm.
